# A novel bioassay for thyroid-blocking immunoglobulins

**DOI:** 10.3389/fendo.2024.1463379

**Published:** 2024-10-29

**Authors:** Augustine George, Johannes Lotz, Maximilian Luffy, Anna-Lena Ganz, Jan Wolf, George J. Kahaly

**Affiliations:** ^1^ Molecular Thyroid Research Lab, Department of Medicine I, Johannes Gutenberg University (JGU) Medical Center, Mainz, Germany; ^2^ Institute of Clinical Chemistry and Laboratory Medicine, Johannes Gutenberg University (JGU) Medical Center, Mainz, Germany

**Keywords:** thyroid-blocking immunoglobulins, thyrotropin receptor blocking antibodies, blocking TSH-R bioassay, homogeneous cAMP biosensor, autoimmune thyroid disease

## Abstract

**Background:**

Thyroid-blocking immunoglobulins (TBI) are present in 10%–15% of patients with autoimmune thyroid disease (AITD). TBI affect thyroid function. The analytical performance of a novel TBI bioassay was evaluated.

**Methods:**

Sera from AITD patients were tested with a cell-based TBI reporter bioassay (Thyretain^®^) with the expression of a luciferase transgene as readout and a new “Turbo™” TBI bioassay with a readout based on a cyclic AMP-activated luciferase. All samples were also run on two TSH-R binding immunoassays. A Passing–Bablok regression, a Bland–Altman plot, and user/lot comparisons were performed. In addition, dose–response curves for Turbo and Thyretain were fitted using serial dilutions, and half-maximal and 80% inhibitory concentrations (IC_50_/IC_80_) were compared.

**Results:**

Of 1,011 unselected AITD patients, 131 patients (212 samples) were TBI positive. Of the 212 samples, 149 (70.3%), 47 (22%), and 16 (7.5%) were hypothyroid, euthyroid, and hyperthyroid, respectively. The three thyrotropin receptor antibody (TSH-R-Ab) assays were negative in 90 controls devoid of autoimmune thyroid disorders. In contrast, the Turbo cyclic adenosine 3′,5′-monophosphate (cAMP) TBI, Thyretain TBI, and the binding assays detected TBI in 212 (100%), 168 (79%), and 138/180 (65%) samples, respectively (*p*< 0.001). Turbo highly correlated with thyroid function (*p*< 0.001). The percentage inhibition in both Turbo and Thyretain correlated with TSH-R-Ab binding assay positivity (both *p*< 0.001). The two bioassays correlated (*r* = 0.8, *p*< 0.001), and the Bland–Altman plot displayed no significant bias (0.24). Values scatter with slight systemic deviation between TBI mean values of 10%–50% inhibition, with higher Turbo than Thyretain results. Intra-assay validation demonstrated adequate precision with a very low coefficient of variation (average CV 5.4%) and lower CV with samples with a high inhibitory effect (CV_Average_= 1.7% for a sample with 95% inhibition Thyretain). CV did not differ between users (*p* = 0.35) and lots (*p* = 0.121). The IC_50_/IC_80_ values were 1.55 ng/mL/3.48 ng/mL for Turbo and 6.76 ng/mL/18.46 ng/mL for Thyretain, respectively, demonstrating the markedly higher sensitivity of Turbo.

**Conclusions:**

The novel, easy-to-perform, rapid, and reliable Turbo TSH-R blocking bioassay detected significantly more TBI than the established immunoassays, emphasizing its higher analytical performance and clinical utility in the management of patients with AITD.

## Introduction

Autoimmune thyroid diseases (AITD) are the most frequent autoimmune disorders (1) and are prevalent in middle-aged women (2, 3). Both autoimmune Hashimoto’s thyroiditis (HT) and Graves’ disease (GD) cause thyroid dysfunction, resulting in hypo- or hyperthyroidism (4). Recent European guidelines for the management of Graves’ hyperthyroidism and Graves’ associated extra-thyroidal manifestations (5, 6) recommend a precise evaluation of clinical manifestations and serological parameters, e.g. thyroid-related hormones and autoantibodies, which are crucial for an effective treatment. In AITD, thyrotropin receptor (TSH-R) autoantibodies (TSH-R-Ab) or pathogenic immunoglobulins targeting the TSH-R are pivotal, disease-specifc and show variable functionality, e.g., stimulatory (TSI) or blocking (TBI), affecting thyroid cell metabolism differently (7–9). TSI can be observed in the newborns of mothers with hypothyroid HT (10), while TBI have been reported in the offspring of mothers with Graves’ hyperthyroidism (11). Furthermore, a shift from TSI to TBI and *vice versa* has been observed in approximately 10% of GD patients during antithyroid drug (ATD) therapy (12).

TBI are present in 10%–15% of patients with AITD (13) and affect thyroid function. In comparison, TSI engage with the large extracellular amino-terminal segment of the TSH-R, leading to the activation of the G-protein-coupled pathway (7). This activation induces a rise in cyclic adenosine 3′,5′–monophosphate (cAMP), leading to an increased synthesis of triiodothyronine (T3) and thyroxine (T4). Additionally, it promotes the proliferation of thyroid follicular endothelial cells, thereby stimulating the growth of the thyroid gland. In contrast, TBI reduce thyrotropin stimulation by competitively obstructing the TSH-R, resulting in decreased thyroid hormone synthesis and cell proliferation. This mechanism may contribute to the hypothyroidism observed in AITD patients (14, 15). The third category of antibodies, known as neutral Ab or “cleavage” Ab, does not stimulate or hinder TSH-R function but can activate alternative pathways, some of which are also triggered by TSI. Through potential G-protein activation, neutral Ab may initiate signaling cascades involving the activation of mammalian target of rapamycin (mTOR), protein kinase C/mitogen-activated protein kinase (MAPK), nuclear factor kappa-light-chain-enhancer of activated B-cells (NF-kB), reactive oxygen species (ROS), and a variety of cytokines. However, to date, the complete clinical and pathological implications of neutral Ab remain unclear. Exposure of rat thyrocytes to neutral Ab resulted in an increased expression of various oncogenes (p53, p73, and retinoblastoma protein), endoplasmic reticulum stress protein (grp98), and heat shock proteins (p27 and p107), ultimately leading to apoptosis (16).

In this study, a novel cAMP-based assay named “Turbo™” TBI is under development, promising reduced complexity, shorter processing time, and an increased sample capacity per run, which could potentially enhance clinical diagnostics. Analyses pertaining to the analytical performance and clinical relevance of this new TBI bioassay are described.

## Materials and methods

This study was conducted in compliance with good clinical practice (GCP) and the local institutional review board. All AITD patients and healthy control subjects provided informed consent prior to blood collection following the tenets of the Declaration of Helsinki.

Serum samples from well-documented AITD patients were tested with a European Conformity (CE)-marked cell-based blocking reporter bioassay (Thyretain^®^ TBI, QuidelOrtho Corporation, San Diego, CA, USA), with the expression of a luciferase transgene as the readout and a new, rapid, and sensitive “Turbo™” TBI bioassay (QuidelOrtho) with a readout that is based on a cAMP-activated luciferase (17). All patients displayed clinical or serological symptoms of AITD, e.g., autoimmune-induced hypothyroidism and goiter. Thyroid function was determined by the measurement of thyroid-related hormones TSH, free-T4, and free-T3 in the serum. A total of 180 serum samples were also run on two TSH-R binding immunoassays (Cobas e411, Roche, Germany, and ALINITY I Immunoassay-System, Abbott, Germany) according to the manufacturer’s instructions. Thyroperoxidase (TPO) and thyroglobulin (Tg) antibodies were measured in 139 and 135 samples, respectively (Cobas e411, Roche, Germany). All samples were tested in duplicate, and percentage inhibition was calculated by comparing the average relative light units from the subjects’ sera with an average reference value (**Supplementary Formula S1**). A Passing–Bablok regression a Bland–Altman plot and user and lot comparisons, were performed with the Turbo™ and Thyretain^®^ TBI cell-based bioassays. In addition, dose–response curves were fitted for both Turbo™ and Thyretain^®^ TBI via serial dilution. A dose–response of the commercially available, purely human TSH-R blocking monoclonal antibody (mAb) K1-70 (RSR, Cardiff, UK) was performed with both TBI bioassays. For this purpose, a K1-70 starting solution with a concentration of 1.3 mg/mL was diluted in reaction buffer (RB) to 17 dilutions ranging from 8,000 to 0.1225 ng/mL for Turbo™ TBI. For Thyretain^®^ TBI, 16 dilutions with concentrations ranging from 363.63 to 0.011 ng/mL were utilized. The IC_50_ and IC_80_ values were determined and compared.

### GS-22F biosensor development

The Turbo™ TBI bioassay employs TSH-R- chimeric Chinese ovarian hamster cells (Mc4 cells) integrated with the pGloSensor™-22F cAMP (GS-22F) biosensor (Promega, Madison, WI, USA) to accurately measure intracellular cAMP levels called homogeneous cAMP biosensor cells (HCBS-TSH-R-Mc4 cells). The GS-22F biosensor differs from its predecessor in that it has five distinct amino acids at the N-terminus of the luciferase unit and six different amino acids in the binding peptide. This results in a fourfold reduction in *in vitro* sensitivity but offers a broader linear measurement range. This modification allows for better differentiation between full and partial agonists across various cell types. In comparative studies, the GS-22F sensor exhibited similar signal strength and response kinetics to a highly sensitive enzyme-linked immunosorbent assay. The GS-22F biosensor utilized in the Turbo™ TBI bioassay represents a further advancement over the previously employed “fluorescence resonance energy transfer” and “bioluminescence resonance energy transfer” cAMP biosensors (18). The GS-20F sensor, an earlier iteration, comprises the cAMP-binding domain “regulatory subunit type IIβ” (RIIβB) from protein kinase A (PKA) and the luciferase active center from *Photinus pyralis*. This sensor exhibits high sensitivity but rapid saturation of the response curve in Human Embryonic Kidney 293 (HEK293) cells. The CE-marked blocking Thyretain^®^ TBI bioassay was used as a control assay and was performed as previously described according to the manufacturer’s instructions (14, 19–21).

### Statistical analysis

The results of the blocking Turbo™, Thyretain^®^, and binding TSH-R-Ab immunoassays were compared using Spearman’s correlation coefficient. The results were visualized in a Passing–Bablok regression considering the diagnosis. The sensitivity and specificity of the Thyretain^®^ TBI and Turbo™ TBI were plotted on a receiver operating curve (ROC), and the area under the curve (AUC) was calculated. The correlation between assay results and thyroid function was analyzed using the Mann–Whitney *U* (MWU) test (22). The effect size *r* values of the MWU were determined using the *Z* value. The *r* values were interpreted according to Cohen’s guidelines (23). The precision of the Turbo™ bioassay was determined using the mean value and coefficient of variation (CV). The mean values of the measurement days and the CV of the two users were compared using a paired *t*-test. Two lots were tested, and the results and CV were compared with an unpaired *t*-test. The results were plotted on a diagram. The Turbo™ TBI measured the dilution levels in two replicates, and the mean value of relative light units (RLU) was plotted graphically using GraphPad Prism 10 (GraphPad Software, Boston, MA, USA). The significance level (*α*) was set at 0.05. Correlations and graphs were created using IBM^®^ SPSS^®^ Statistics 23 (Armonk, New York) and MedCalc 20.118 (MedCalc Software Ltd., Ostend, Belgium).

## Results

### Demographic and clinical data

One thousand eleven (*n* = 1,011) unselected, consecutive AITD patients with a median disease duration of 5 years, range 0–7.5 years, were enrolled. Of these 1,011 subjects, 131 patients (median age, 33 years; 25–75th percentile, 13–47.5 years; female/male ratio, 2.9:1) and 212 corresponding samples were TBI positive. Of the 212 samples, 149 (70.3%), 47 (22%), and 16 (7.5%) were hypothyroid, euthyroid, and hyperthyroid, respectively. When drawing blood, 52 and nine AITD patients were on levothyroxine (L-T4) and ATD, respectively. A total of 90 healthy subjects (54 female) devoid of autoimmune thyroid and endocrine disorders served as controls.

### Serology

**Figure 1:**
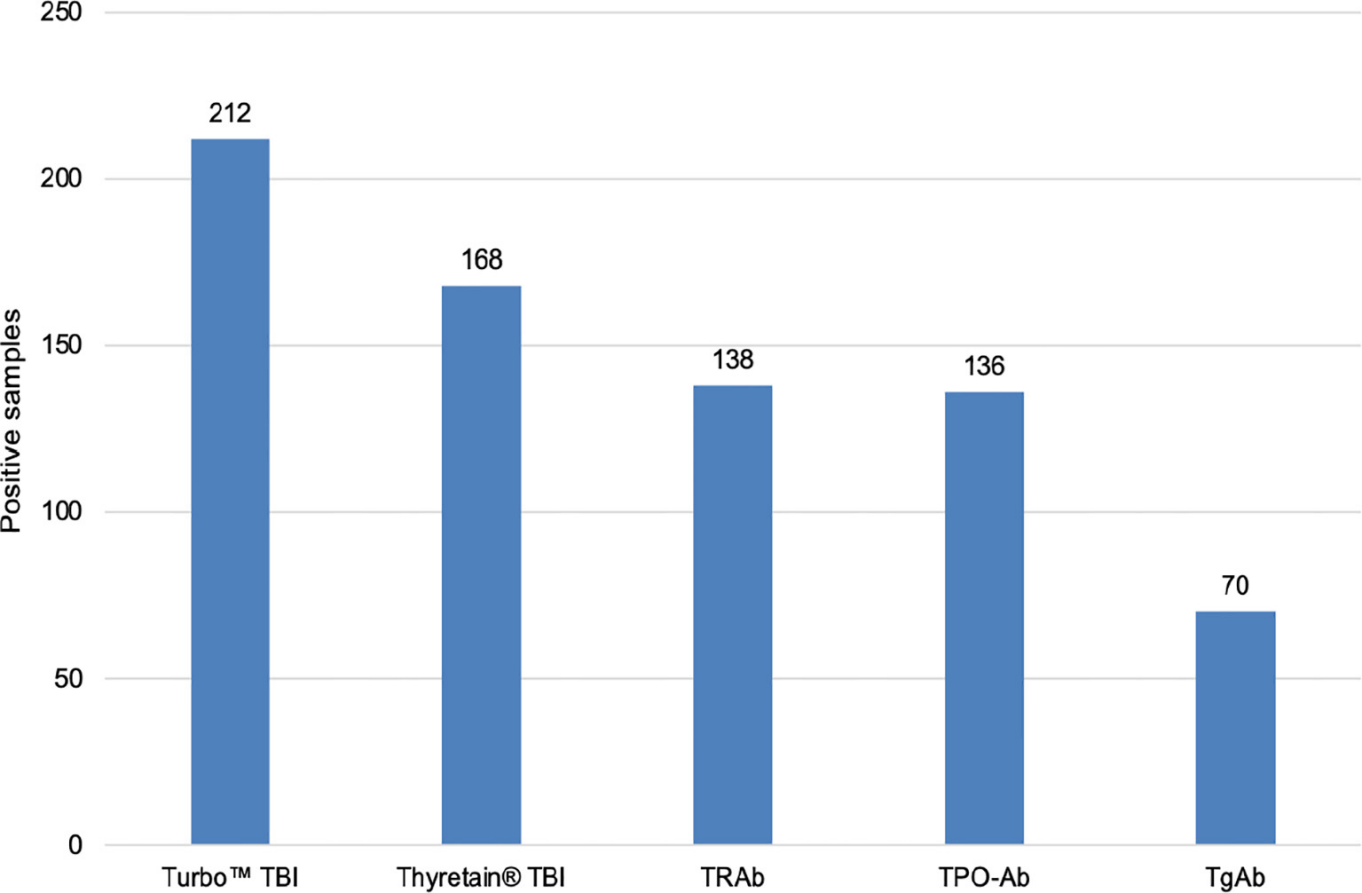
Number of TBI-positive samples in various serological tests. *N* = 212 samples and 131 patients. Cutoff values: Turbo™ TBI >40 percentage inhibition; Thyretain^®^ TBI >34 percentage inhibition.

**Figure 2:**
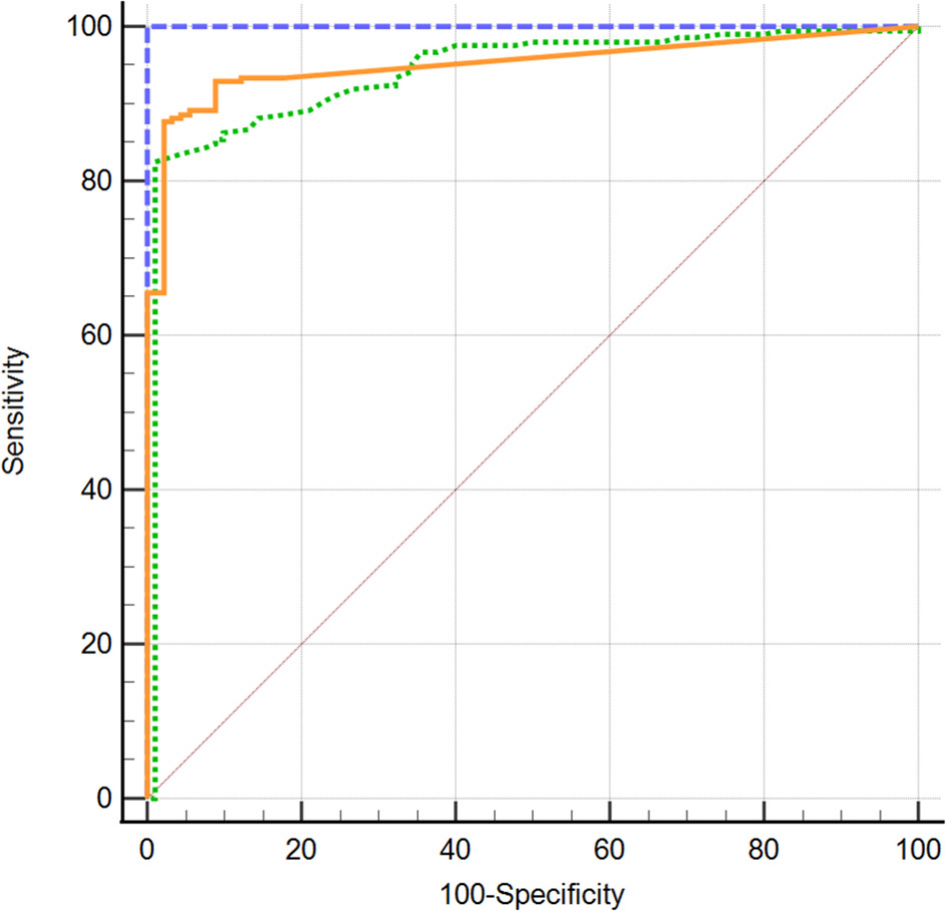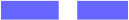 Turbo™ TBI 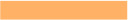 Thyretain^®^ TBI 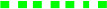 TRAb immunoassay. ROC diagram of 302 samples (212 AITD and 90 controls) tested with Turbo™, Thyretain^®^ TBI and TRAb immunoassay. AUC (95% CI): Turbo™ 1,000 (0,986 - 1,000), Thyretain^®^ 0,950 (0,919 -0,972), and TRAb 0.952 (0.921-0.973).

All three TSH-R-Ab assays were negative in all 90 controls. A total of 31 and 49 samples displayed negative percentage inhibition in the Turbo™ and Thyretain^®^ TBI, as the average relative light units of the subjects were higher than the reference value (**Supplementary Formula S1**). In contrast, the Turbo™ cAMP TBI, the Thyretain^®^ luciferase TBI, and the binding assays detected TBI in 212 (100%), 168 (79%), and 138/180 (65%) samples, respectively (**Figure 1**). Serum TSH-R-Ab positivity was concordant in only 134 of the 212 samples, or 63.2% (**Table 1**). **Figure 2** compares both TBI bioassays on the ROC diagram. Turbo™ displays both sensitivity (95% CI, 98.28%–100%) and specificity (95.98%–100%) levels of 100%. Thyretain^®^ shows a sensitivity level of only 79.25% (73.16-84.5%) and a specificity level of 100% (95.98%–100%). In contrast, the sensitivity of the binding assays was markedly lower (65.09%, 58.27%–71.49%), with a specificity level of 100% (95.98%–100%).

**Table 1:** Concordance of serum TSH-R-Ab positivity in the Turbo™, Thyretain^®^, and binding immunoassays.

Turbo™ TBI	Thyretain^®^ TBI	TRAb	Samples (*N* = 212)	Patients (*N* = 133)
**(+)**	**(+)**	**(+)**	134 (63.2%)	62 (46.6%)
**(+)**	**(+)**	**(-)**	34 (16.0%)	27 (20.6%)
**(+)**	**(-)**	**(+)**	4 (1.9%)	4 (3.0%)
**(+)**	**(-)**	**(-)**	40 (18.9%)	40 (30.1%)

Positive results are indicated in green, while negative results are highlighted in red.

**Figure 3:**
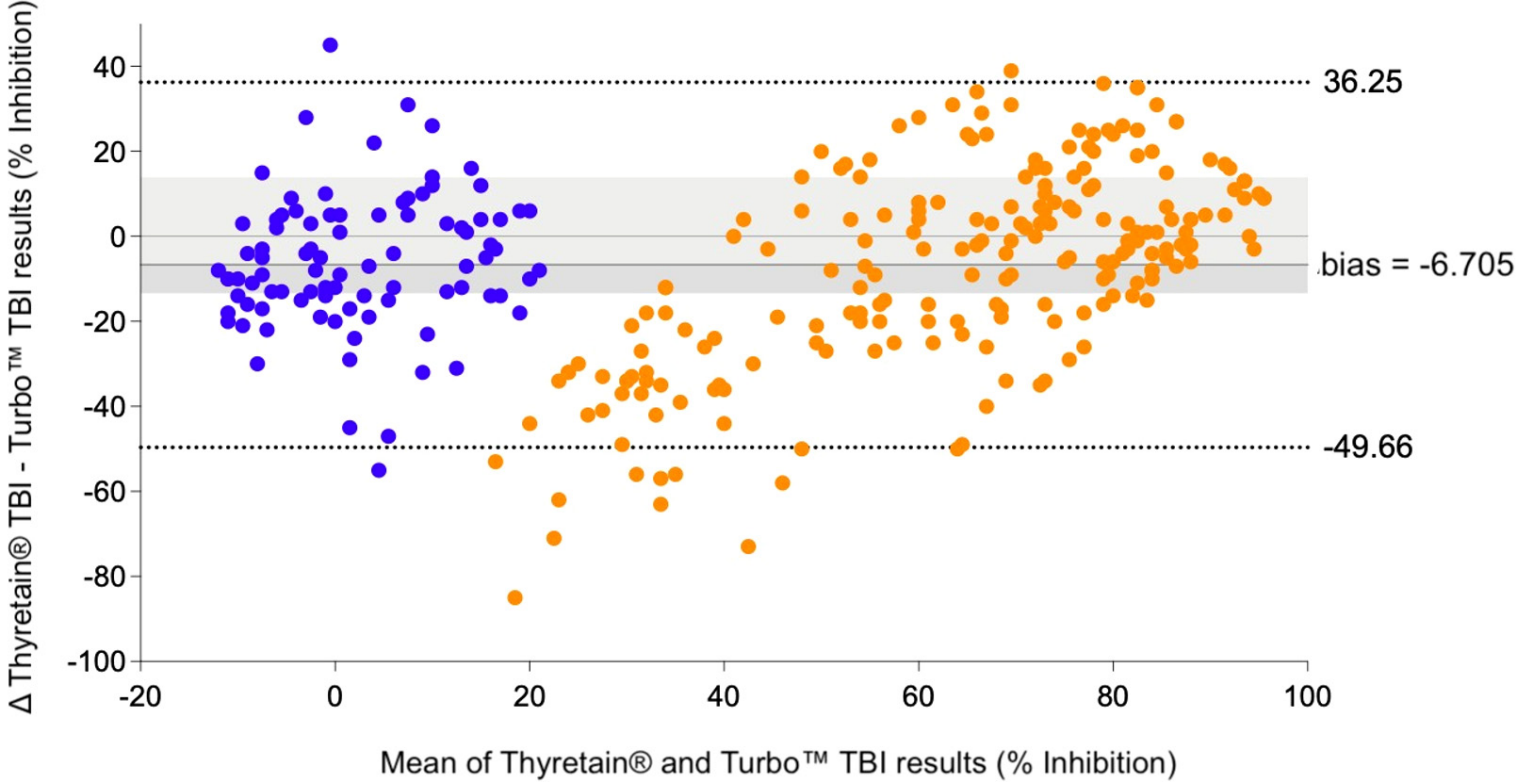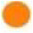, AITD; 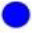, control. Bland–Altman plot of Turbo™ and Thyretain^®^ TBI results.

**Figure 4:**
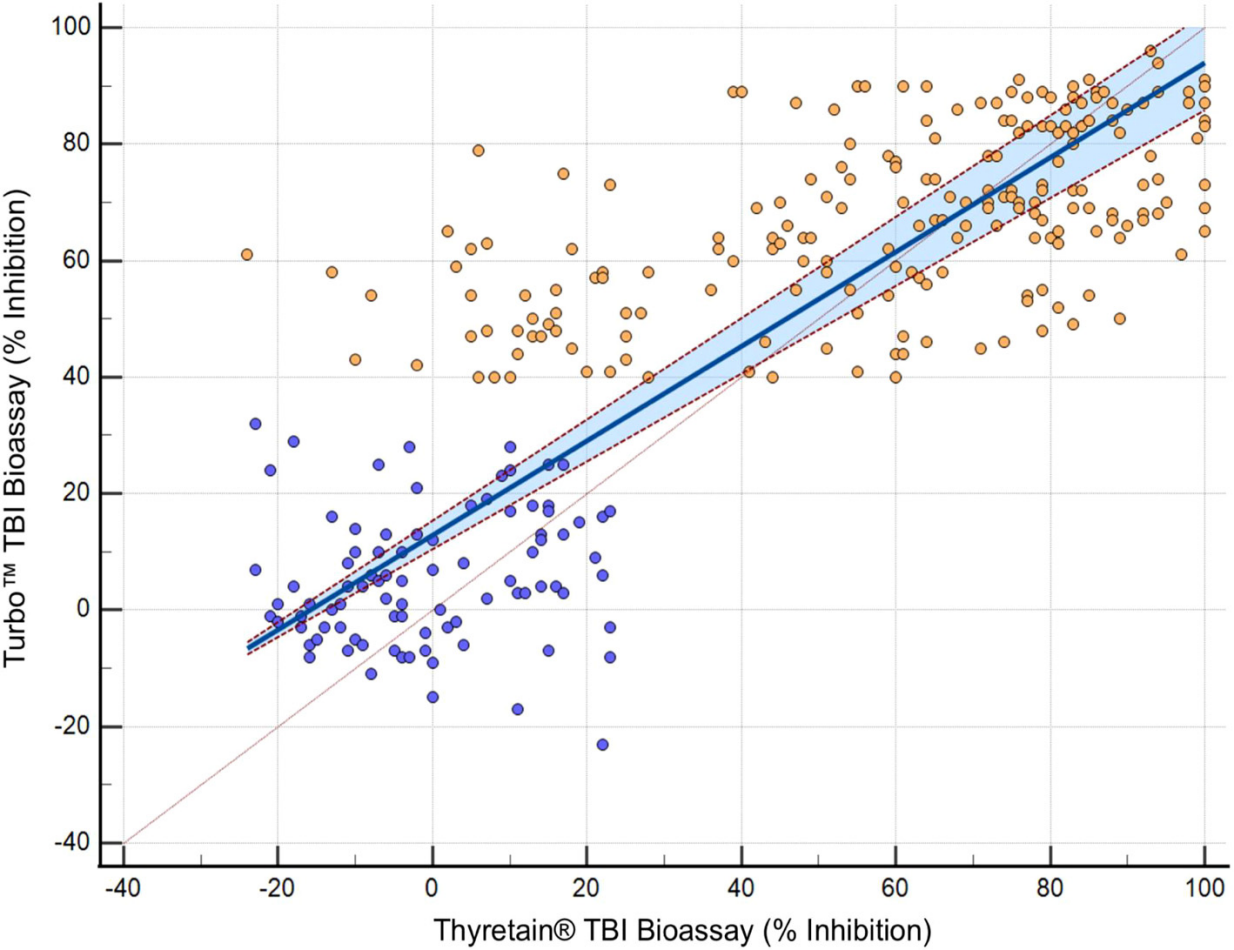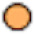, AITD; 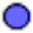, control. Passing–Bablok regression of Turbo™ and Thyretain^®^ TBI results. The blue area marks a 95% confidence interval. *N* = 302 (221 subjects). Spearman’s Rho, 0.8; 95% CI, 0.75–0.84; *p*< 0.001. *y* = 12.878378 + 0.810811*x*.

In the Bland–Altman diagram (**Figure 3**), the values are scattered with a slight systemic deviation between TBI mean values of 10% to 50% inhibition. Within this range, the Turbo™ results are consistently higher than those obtained with Thyretain^®^. Consequently, the mean difference between the paired results is -6.705%, indicating that Turbo™ % inhibition values tend to be higher, on average, compared to Thyretain^®^ values. The Thyretain^®^ and Turbo™ results correlate (Spearman’s Rho_TurboTM_ Thyretain® = 0.8; 95% CI, 0.75–0.84; *p*< 0.001; **Figure 4**). Similar correlations were observed between the results of the binding TRAb and the two bioassays (Spearman’s Rho_TurboTM_TRAb = 0.74, 0.69–0.79, *p*< 0.001; Spearman’s Rho_Thyretain^®^_TRAb = 0.78, 0.69–0.79, *p*< 0.001).

Different values of Turbo™ were noted depending on positive or negative TRAb, TgAb, and TPO-Ab results (*p*< 0.001) (**Supplementary Tables S4A, B**). The effect size *r* was strong for TRAb and TPO-Ab (*r*_TRAb_ = 0.676, *r*_TPO-Ab_ = 0.580), while it was weak for Tg-Ab (*r*_Tg-Ab_ = 0.262). In comparison, Thyretain^®^ showed comparable associations with TRAb (**Table 2B**), Tg-Ab, and TPO-Ab (*p*< 0.001) with similar effect sizes (*r*_TRAb_ = 0.745, *r*_TPO-Ab_ = 0.639, and *r*_Tg-Ab_ = 0.273).

On the other hand, when considering the AITD cohort only, Turbo™ results differed based on whether the TRAb results were positive or negative (*r* = 0.43, *p*< 0.001) (**Table 2A**). While Turbo™ and Tg-Ab or TPO-Ab did not correlate significantly (*p*_Tg-Ab_ = 0.078 and *p*_TPO-Ab_ = 0.147), the Thyretain^®^ results did (Tg-Ab and TPO-Ab: *p*< 0.001, *r*_Tg-Ab_ = 0.05 and *r*_TPO-Ab_ = 0.31). Samples with low Turbo™ TBI positivity (40%–50% inhibition) showed fewer TPO-Ab positive samples (64.3%), while the proportion of TPO-Ab positive samples increased (79.7%) in the high Turbo™ TBI positive samples (71%–100% inhibition). Similarly, the proportion of Tg-Ab positive samples increased when comparing low vs. high percentage inhibition ranges (32.4% vs. 42.2%). TPO-Ab and Tg-Ab show a similar distribution in the low (34%–50%) and high (71%–100%) inhibition ranges of Thyretain^®^ TBI (TPO-Ab 52.6 vs. 89.6% and Tg-Ab 33.3 vs. 44.4%).

**Table 2A:** Relationship between Turbo™ TBI, Thyretain^®^ TBI, and TRAb results.

Turbo™ TBI reporter bioassay inhibition range (% inhibition)	40–50 *N* = 38	51–60 *N* = 32	61–70 *N* = 53	71–100 *N* = 89	Total *N* = 212
Thyretain^®^ TBI positive	15 (39.5%)	19 (59.4%)	48 (90.6%)	86 (96.7%)	168 (79.2%)
TRAb positive	14 (36.8%)	10 (31.3%)	39 (73.6%)	75 (84.3%)	138 (65.1%)

TBI ranges sort the assay results. Turbo™ TBI values were tested using the Mann–Whitney *U* (MWU) test: *p*_TRAb_ *<*0.001; TRAb shows moderate strength (*r*_TRAb_ = 0.43).

**Table 2B:** Relationship between Thyretain^®^ TBI and TRAb results.

Thyretain^®^ TBI inhibition range (% inhibition)	34–50 *N* = 21	51–60 *N* = 22	61–70 *N* = 23	71–100 *N* = 102	Total *N* = 168
TRAb positive	7 (33.3%)	14 (63.3%)	20 (87.0%)	93 (91.2%)	134 (79.8%)

TBI ranges sort the assay results. MWU tests were repeated with Thyretain^®^ TBI (*p*_TRAb_ *<*0.001); TRAb shows robust (*r* = 0.61) strength.

Contingency tables (**Tables 3A**, **B**) show the distribution of functional antibodies in hypothyroid, euthyroid, and hyperthyroid sera. The results of both TBI bioassays differed depending on whether the samples were hypo- or non-hypothyroid (*p*< 0.001), with a slightly higher effect size of Turbo™ compared to Thyretain^®^ (*r*_TurboTM_TBI = 0.576 *vs. r*_Thyretain^®^TBI_ = 0.51). Turbo™ (in contrast to Thyretain^®^, *p* = 0.08) exhibited significant differences (*p* = 0.03, *r* = 0.15) between hypo- and non-hypothyroid samples when only the autoimmune AITD cohort was considered.

**Table 3A:** Relationship between Turbo™ results and thyroid function (Fisher’s exact test, *p* = 0.121; AITD patients only).

Turbo™ TBI inhibition range (% inhibition)	40–50	51–60	61–70	71–100	Total
Samples	38	32	53	89	212
Hypothyroid	20 (**52.6%**)	21 (**65.6%**)	40 (**75.5%**)	68 (**76.4%**)	149 (**70.3%**)
Euthyroid	14 (**36.8%**)	7 (**21.9%**)	9 (**17.0%**)	17 (**19.1%**)	47 (**22.2%**)
Hyperthyroid	4 (**10.5%**)	4 (**12.5%**)	4 (**7.5%**)	4 (**4.5%**)	16 (**7.5%**)

Serum samples are sorted by TBI titer. Bold values represent the percentage of hypo-, eu-, and hyperthyroid samples within the respective inhibition range.

**Table 3B:** Relationship between Thyretain^®^ results and thyroid function (Fisher’s exact test, *p* = 0.025; φ 0.203; AITD patients only).

Thyretain^®^ TBI reporter bioassay inhibition range (% inhibition)	34–50	51–60	61–70	71–100	Total
Samples	21	22	23	102	168
Hypothyroid	12 (**54.5%**)	10 (**45.5%**)	18 (**78.3%**)	80 (**78.4%**)	120 (**71.4%**)
Euthyroid	6 (**28.6%**)	10 (**45.5%**)	4 (**17.4%**)	16 (**15.7%**)	36 (**21.4%**)
Hyperthyroid	3 (**14.3%**)	2 (**9.1%**)	1 (**4.3%**)	6 (**5.9%**)	12 (**7.1%**)

Serum samples are sorted by TBI titer. Bold values represent the percentage of hypo-, eu-, and hyperthyroid samples within the respective inhibition range.

**Supplementary Figure S1** compares the ability of Turbo™ and Thyretain^®^ to differentiate between hypo- and non-hypothyroid samples. The area under the curve (AUC) of Turbo™ is greater than that of Thyretain^®^ (Turbo™ 0.833, 0.786–0.873 vs. Thyretain^®^ 0.795, 0.745–0.839; *p* = 0.0616). Furthermore, Turbo™ showed higher sensitivity with cutoffs between false-positive rates of 20%–80% (**Supplementary Figure S1**).

**Figure 5:**
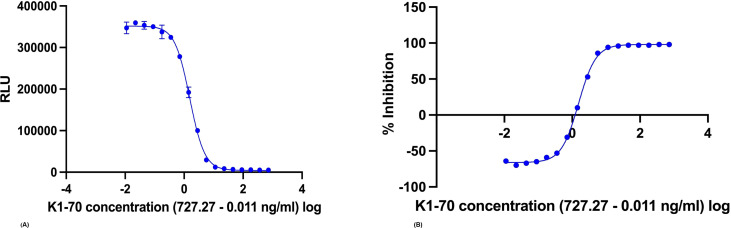
**(A)** Dose–response curve by measuring a K1-70 dilution series with the Turbo™ TBI bioassay (IC_50_ = 1.55 ng/mL, log (IC_50_) = 0.19; IC_80_ = 3.48 ng/mL, log (IC_80_) = 0.54). **(B)** Dose–response curve by measuring a K1-70 dilution series with the Thyretain^®^ TBI bioassay (IC_50_ = 6.76 ng/mL, log (IC_50_) = 0.83; IC_80_ = 18.45 ng/mL, log (IC_80_) = 1.27).

**Figure 6:**
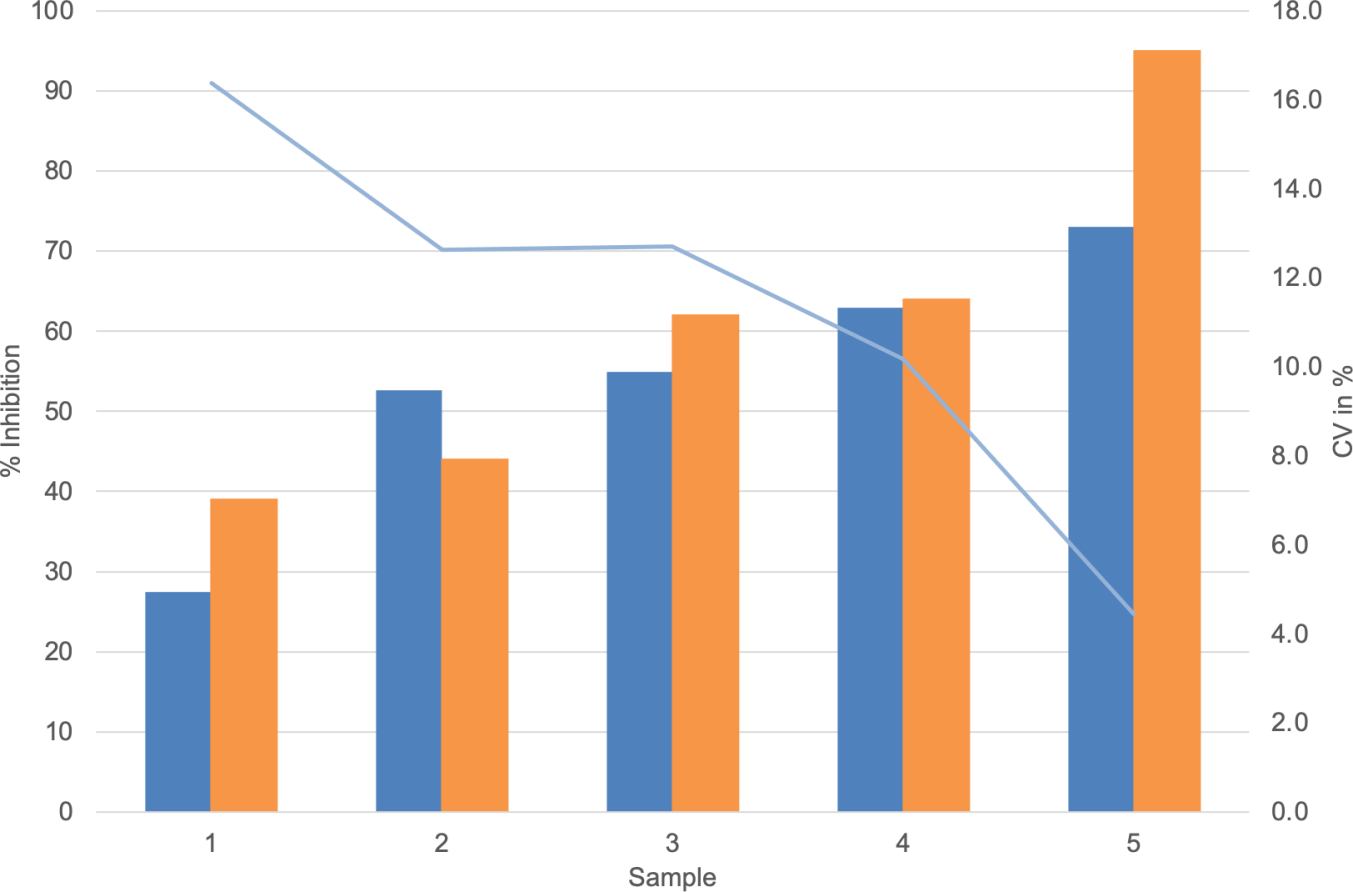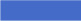, Average Turbo™ TBI values; 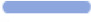, CV Turbo™ TBI; 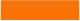, Average Thyretain^®^ TBI values. Mean value of the Turbo™ TBI precision measurements with associated CV and the Thyretain^®^ TBI initial value of the respective sample. Cutoff values: Turbo™ TBI > 40 percentage inhibition; Thyretain^®^ TBI > 34 percentage inhibition. The average inhibition values are 59.45% (SD 54.15%) for user 1 and 54.15% (SD 16.72%) for user 2, with average CVs of 6% (SD 0.0492) for user one and 8% (SD 0.0735) for user 2 (*p* = 0.32). There was no statistical difference between Turbo™ and Thyretain^®^ values: Thyretain^®^ had an average inhibition of 60.8% (SD 22.02) and Turbo™ had an inhibition of 54.20% (SD, 16.92) (*p* = 0.27).

**Figure 7:**
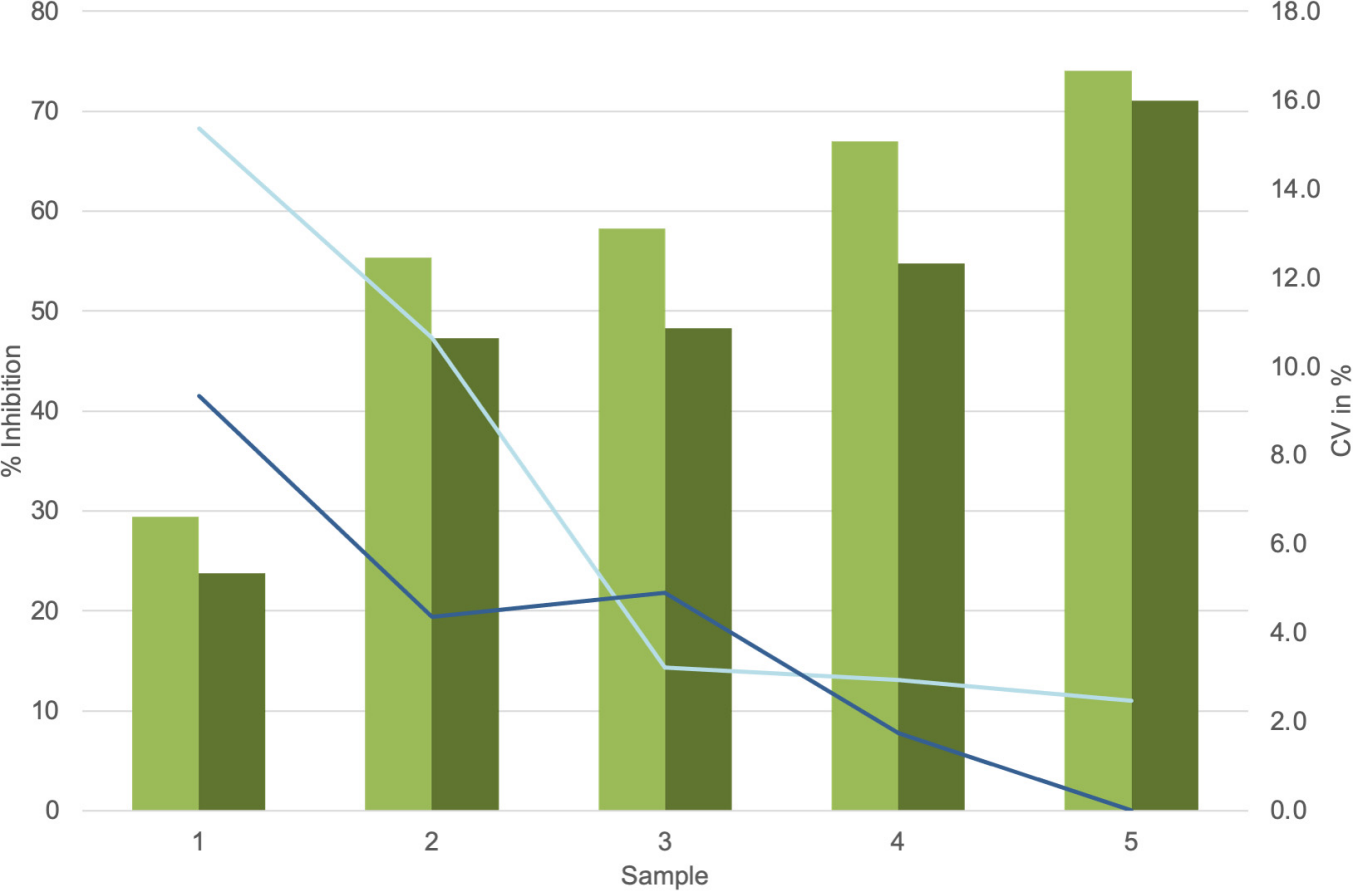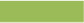, Average values lot 1; 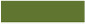, Average values lot 2; 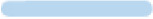, CV lot 1; 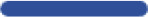, CV lot 2. Presentation of the Turbo™ TBI mean values and CV. Cutoff values: Turbo™ TBI >40 percentage inhibition, Thyretain^®^ TBI >34 percentage inhibition. For the respective lots, the average inhibition values are 56.8% (SD 16.06%) for lot 1 and 49.0% (SD 15.69%) for lot 2, with no significant difference (*p* = 0.08). The CV is 7% (SD, 0.06) for lot 1 and 4% (SD, 0.03) for lot 2 (*p* = 0.34).

Dose–response curves using the K1-70 blocking mAb gave IC_50_/IC_80_ of 1.55 ng/mL/3.48 ng/mL for Turbo™ (**Figures 5A, B**) and 6.76 ng/mL/18.45 ng/mL for Thyretain^®^ (**Supplementary Figures S2A, B**). Intra-assay validation demonstrated adequate precision with very low CVs (average, 5.4%) for TBI-positive samples and lower CV values for samples with a high inhibitory effect (**Figure 6**). The CV did not differ between users (*n* = 2, *p_χ_*_²_ = 0.35) or between lots (*n* = 2, *p_χ_*_²_ = 0.121) (**Figure 7**, **Supplementary Tables S1**–**S3**).

## Discussion

The novel, easy-to-perform, rapid, and reliable Turbo™ TSH-R blocking reporter bioassay reproducibly detected significantly more TBI than the established conventional binding and cell-based assays for TSH-R-Ab measurement. This emphasizes its higher analytical performance and clinical utility in the management of patients with AITD, e.g., GD and HT. Good precision data with low variability of results between users and lots were also demonstrated.

A one-to-one comparison between new (Turbo™) and current (Thyretain^®^) assays emphasizes the significant progress achieved. Indeed Turbo™ has several significant advantages over Thyretain^®^ (**Table 4**). An important advantage is the use of all wells on the assay plate. This means that 90 samples can be measured in the Turbo™ TBI assay as opposed to only 21 samples in the current Thyretain^®^ TBI assay. The cells for Turbo™ are ready to use after thawing. Thus, an incubation time of 16h-18h, which is warranted for Thyretain^®^ is not required. Furthermore, there is no need for an incubator and/or aseptic conditions. Sample dilution in the current Thyretain^®^ TBI bioassay requires approximately 1 h and several dozen Eppendorf tubes. With Turbo™, patient sera can be pipetted into the wells without dilution. Only 10 µL of the patient’s serum is required with Turbo™ *vs* 30 µL of serum for Thyretain^®^. Due to this lower sample volume, electronic pipettes for the Turbo™ bioassay are recommended, thus allowing easier handling and reduced susceptibility to errors during pipetting.

**Table 4:** Comparison of the novel Turbo™ cell-based TBI reporter bioassay with the established, CE-marked Thyretain^®^ TBI reporter bioasay.

Assay characteristics	Thyretain^®^ TBI bioassay	Turbo™ TBI bioassay
Platform	Cell-based cell culture lytic bioluminescent assay	Cell-based real-time, non-lytic bioluminescent assay
Reporting pathway	Luciferase reporter gene	cAMP reporter
Results	% inhibition	% inhibition
PPV/NPV	94%/90%	99%/95%
Samples	21 patient samples per plate	90 patient samples per plate
Sample volume	30 µL serum required	10 µL serum required
Assay temperature	37°C/5% CO_2_	Room temperature
Cell incubation time	Seeding and incubating of cells for 15–18 h	No need to incubate the cells
Serum incubation time	3 h time for the cells to react with sera	1 h time for the cells to react with sera
Assay time	~20 h	~2 h
Clearance	CE marked	CE marked
Feasibility	High-complexity labs	Moderate-complexity labs

Further advantages of the Turbo™ bioassay result from the use of HCBS-TSH-R-Mc4 cells, which are produced by transfecting linearized GS-22F and TSH-R-Mc4 plasmid into Chinese Hamster Ovary K1 cells. The selected sub-clones of the transfected cells are referred to as HCBS-TSH-R-Mc4 (17). The results of the measurements of bovine TSH (bTSH) and the commercially available human stimulatory monoclonal TSH-R-Ab M22 indicated that there were differing responses between HCBS-TSH-R-Mc4 and wild-type (wt.) Mc4 cells. The response of bTSH was found to be lower in HCBS-TSH-R-Mc4 cells compared to Wt-Mc4 cells (signal/control ratio: Wt-Mc4 = 24.7 vs. HCBS-TSH-R-Mc4 = 12.1). Conversely, M22 experiments revealed a higher response in HCBS-TSH-R-Mc4 cells (signal/control ratio: Wt-Mc4 = 28.0 vs. HCBS-TSH-R-Mc4 = 40.6). Furthermore, the study demonstrated comparable analytical performance between Turbo™ TSI and Thyretain^®^ TSI, with high Precision (CV < 15%) and EC_50_ values of 4.7 and 5.5 ng/mL, respectively, using M22. The Turbo™ TSI demonstrated high sensitivity (98.7%) and specificity (93.5%) and exhibited no cross-reactivity with 22 substances, including hormones, drugs, and antibodies, when tested at high doses (1,000 ng/mL). K1-70 did not produce false-positive results but did reduce light signals induced by bTSH. With these promising results in the Turbo™ TSI, the authors concluded a possible use case for a blocking-type bioassay (Turbo™ TBI bioassay) (17).

Overall, the Turbo™ TBI results correlated better with hypothyroid patient samples than Thyretain^®^. Agreement of the ROC diagram is observed with cutoff values above a false-positive rate of 20%. In line with this, the effect size for Turbo™ TBI to detect hypothyroid samples was higher than the effect size for Thyretain^®^. In AITD, Turbo™ showed significantly different results depending on TRAb positivity and hypothyroid samples. In contrast to Turbo™, the Thyretain^®^ results did not differ between hypothyroid and non-hypothyroid samples. Overall, Turbo™ was better at recognizing hypothyroid patients than Thyretain^®^. In both bioassays, the proportion of hypothyroid patients increased with the range of percentage inhibition, while the number of euthyroid and hyperthyroid samples markedly decreased. This provides further evidence of TBI influencing thyroid function. In detail, the number of hypothyroid samples detected with Turbo™ TBI increased gradually with each percentage inhibition range, while the number of TBI-positive samples increased suddenly in the high-positive range with Thyretain^®^.

Measuring functional antibodies during pregnancy in AITD patients may be vital, as previous publications have reported fetal thyroid dysfunction due to transferred maternal functional TSH-R-Ab. This can be unexpected, e.g., a previously reported neonatal hypothyroidism in a newborn born to a mother diagnosed with GD (10). Moreover, TBI-induced transient hypothyroidism in newborns has been frequently reported (14, 24, 25). Therefore, current guidelines for the management of pregnant subjects with AITD, e.g., Graves’ disease and/or HT, recommend the measurement of functional antibodies (6).

The significant serological differences pertaining to TSH-R-Ab positivity and thyroid dysfunction, especially in hypothyroidism, underline the clinical relevance of these tests. Indeed, a detailed analysis of functional antibody distribution in various inhibition ranges suggests that disease activity correlates with the presence of blocking TSH-R-Ab. In comparison, TPO-Ab is a marker for the presence of AITD. In contrast, Tg-Ab may be absent in the majority of AITD patients. Tg-Ab is also significantly less prevalent than TPO-Ab in AITD patients. This was the rationale for the contrasting correlation between the two mentioned antibodies (**Supplementary Tables S4A, B**).

The AITD cohort included 11 and 116 patients on antithyroid drugs and thyroid hormones, respectively. No influence of the medication taken on bioassay performance was noted. Furthermore, in a previous report (17), both levothyroxine and liothyronine were incubated with samples in the Turbo™ bioassay and did not interfere with the results of the novel assay. In daily practice, no discrepancies were observed with either bioassay. Based on our current understanding, cross-reactivity with antithyroid or thyroid medications is also not expected.

Based on the obtained data, the implementation of the Turbo™ TBI bioassay in clinical settings could have a significant impact on the diagnosis and monitoring of AITD. The higher sample capacity and the immediate readiness of the cells with this rapid and easy-to-perform assay will save time, workload, and financial resources. The direct application of samples using electric pipettes in prefabricated wells further simplifies the procedure and reduces the susceptibility to error and the complexity of determining functional Ab.

As a current testing limitation of Turbo™ TBI, we acknowledge potential variability within the low-positive inhibition range. Further investigations and optimizations are ongoing to improve the applicability of this challenging assay in different clinical scenarios. On the other hand, the high concordance of Turbo™ with current immunoassays testifies to its high sensitivity and specificity in the identification of AITD patients. These results support the position of Turbo™ TBI as a promising method for the investigation of thyroid diseases and emphasize its diagnostic advantages. Therefore, despite the general preference of large commercial laboratories for automated TRAb measurement due to its ease of performance compared to cell-based bioassays, Turbo™ TBI offers a good alternative due to its ease of use. Its potential integration and implementation into routine clinical testing is likely to lead to a more accurate, effective, and accessible diagnosis of AITD in general and HT in particular.

## Data Availability

The raw data supporting the conclusions of this article will be made available by the authors, without undue reservation.

## Supplementary material

Supplementary Formula 1 Formula to calculate the percentage inhibition from relative light units in the Turbo™ TBI and Thyretain^®^ TBI bioassays. ${\bar{x}}_{reference}=$ average relative light units of the reference sample duplicate, ${\bar{x}}_{sample}=$ average relative light units of the patient sample duplicate.

Supplementary Figure 1
 Turbo™ TBI  Thyretain^®^ TBI. ROC diagram of 302 hypothyroid and non-hypothyroid samples (212 AITD cohort and 90 controls) tested with Turbo™ and Thyretain^®^ TBI. AUC (95% CI): Turbo™ TBI 0.833 (0.786–0.873); Thyretain^®^ TBI 0.795 (0.745–0.839).

Supplementary Figure 2
**(A, B)** Dose–response curve by measuring a K1-70 dilution series with the Thyretain^®^ TBI bioassay, IC_50_ = 6.76, log (IC_50_) = 0.83; IC_80_ = 18.45, log (IC_80_) = 1.72.

Supplementary Table 1 Precision measurement of user 1 (lot 1). Cutoff values: Turbo™ TBI > 40 percentage inhibition; Thyretain^®^ TBI > 34 percentage inhibition.

Supplementary Table 2 Precision measurement of user two (lot 1). Cutoff values: Turbo™ TBI > 40 percentage inhibition; Thyretain^®^ TBI > 34 percentage inhibition.

Supplementary Table 3 Precision measurement of user 1 (lot 2). Cutoff values: Turbo™ TBI > 40 percentage inhibition; Thyretain^®^ TBI > 34 percentage inhibition.

Supplementary Table 4
**(A, B)** Concordance of serum positivity in the Turbo™, Thyretain^®^, and TPO-Ab and Tg-Ab.

## References

[1] Davies TF Andersen S Latif R Nagayama Y Barbesino G Brito M. Graves’ disease. Nat Rev Dis Primers. (2020) 6:52. doi: 10.1038/s41572-020-0184-y 32616746

[2] Frommer L König J Chatzidou S Chionos G Längericht J Kahaly GJ. Recurrence risk of autoimmune thyroid and endocrine diseases. Best Pract Res Clin Endocrinol Metab. (2023) 37:101636. doi: 10.1016/j.beem.2022.101636 35365417

[3] Buonfiglio F Ponto KA Pfeiffer N Kahaly GJ Gericke A. Redox mechanisms in autoimmune thyroid eye disease. Autoimmun Rev. (2024) 23:103534. doi: 10.1016/j.autrev.2024.103534 38527685

[4] Kahaly GJ. Management of graves thyroidal and extrathyroidal disease: an update. J Clin Endocrinol Metab. (2020) 105:3704–20. doi: 10.1210/clinem/dgaa646 PMC7543578 32929476

[5] Bartalena L Kahaly GJ Baldeschi L Dayan CM Eckstein A Marcocci C. The 2021 European Group on Graves’ orbitopathy (EUGOGO) clinical practice guidelines for the medical management of Graves’ orbitopathy. Eur J Endocrinol. (2021) 185:G43–g67. doi: 10.1530/eje-21-0479 34297684

[6] Kahaly GJ Bartalena L Hegedüs L Leenhardt L Poppe K Pearce SH. European thyroid association guideline for the management of graves’ Hyperthyroidism. Eur Thyroid J. (2018) 7:167–86. doi: 10.1159/000490384 PMC6140607 30283735

[7] Kahaly GJ Diana T Olivo PD. TSH RECEPTOR ANTIBODIES: RELEVANCE & UTILITY. Endocr Pract. (2020) 26:97–106. doi: 10.4158/EP-2019-0363 32022598

[8] Lytton SD Kahaly GJ. Bioassays for TSH-receptor autoantibodies: an update. Autoimmun Rev. (2010) 10:116–22. doi: 10.1016/j.autrev.2010.08.018 20807591

[9] Diana T Ponto KA Kahaly GJ. Thyrotropin receptor antibodies and Graves’ orbitopathy. J Endocrinol Invest. (2021) 44:703–12. doi: 10.1007/s40618-020-01380-9 PMC8310479 32749654

[10] Kiefer FW Klebermass-Schrehof K Steiner M Worda C Kasprian G Diana T. Fetal/neonatal thyrotoxicosis in a newborn from a hypothyroid woman with hashimoto thyroiditis. J Clin Endocrinol Metab. (2017) 102:6–9. doi: 10.1210/jc.2016-2999 27813690

[11] Decallonne B Martens PJ Van den Bruel A Vanhole C Kahaly GJ. Graves disease with thyroid-stimulating hormone receptor-blocking autoantibodies during pregnancy. Ann Intern Med. (2020) 172:767–9. doi: 10.7326/l19-0818 32203974

[12] Kahaly GJ Diana T Kanitz M Frommer L Olivo PD. Prospective trial of functional thyrotropin receptor antibodies in graves disease. J Clin Endocrinol Metab. (2020) 105:e1006–14. doi: 10.1210/clinem/dgz292 PMC7067543 31865369

[13] Olivo PD. Bioassays for thyrotropin receptor autoantibodies. Best Pract Res Clin Endocrinol Metab. (2023) 37:101744. doi: 10.1016/j.beem.2023.101744 36828714

[14] Diana T Olivo PD Kahaly GJ. Thyrotropin receptor blocking antibodies. Horm Metab Res. (2018) 50:853–62. doi: 10.1055/a-0723-9023 PMC6290727 30286485

[15] Adams DD. Pathogenesis of the hyperthyroidism of graves’s disease. Br Med J. (1965) 1:1015–9. doi: 10.1136/bmj.1.5441.1015 PMC2166943 14262190

[16] Morshed SA Ando T Latif R Davies TF. Neutral antibodies to the TSH receptor are present in Graves’ disease and regulate selective signaling cascades. Endocrinology. (2010) 151:5537–49. doi: 10.1210/en.2010-0424 PMC2954721 20844004

[17] Miao LY Kim HJ Whitlatch K Jaiswal D Navarro A Egan R. A rapid homogenous bioassay for detection of thyroid-stimulating antibodies based on a luminescent cyclic AMP biosensor. J Immunol Methods. (2022) 501:113199. doi: 10.1016/j.jim.2021.113199 34871593

[18] Binkowski BF Butler BL Stecha PF Eggers CT Otto P Zimmerman K. A luminescent biosensor with increased dynamic range for intracellular cAMP. ACS Chem Biol. (2011) 6:1193–7. doi: 10.1021/cb200248h 21932825

[19] Li Y Kim J Diana T Klasen R Olivo PD Kahaly GJ. A novel bioassay for anti-thyrotrophin receptor autoantibodies detects both thyroid-blocking and stimulating activity. Clin Exp Immunol. (2013) 173:390–7. doi: 10.1111/cei.12129 PMC3949626 23647395

[20] Diana T Li Y Olivo PD Lackner KJ Kim H Kanitz M. Analytical performance and validation of a bioassay for thyroid-blocking antibodies. Thyroid. (2016) 26:734–40. doi: 10.1089/thy.2015.0447 26956921

[21] Diana T Krause J Olivo PD König J Kanitz M Decallonne B. Prevalence and clinical relevance of thyroid stimulating hormone receptor-blocking antibodies in autoimmune thyroid disease. Clin Exp Immunol. (2017) 189:304–9. doi: 10.1111/cei.12980 PMC5543506 28439882

[22] Nachar N. The mann-whitney U: A test for assessing whether two independent samples come from the same distribution. Tutorials Quantitative Methods Psychol. (2008) 4:14-9. doi: 10.20982/tqmp.04.1.p013

[23] Fritz CO Morris PE Richler JJ. Effect size estimates: current use, calculations, and interpretation. J Exp Psychol Gen. (2012) 141:2–18. doi: 10.1037/a0024338 21823805

[24] Castellnou S Bretones P Abeillon J Moret M Perrin P Chikh K. Congenital hypothyroidism due to a low level of maternal thyrotropin receptor-blocking antibodies. Eur Thyroid J. (2021) 10:174–8. doi: 10.1159/000509015 PMC8077501 33981622

[25] Evans C Jordan NJ Owens G Bradley D Ludgate M John R. Potent thyrotrophin receptor-blocking antibodies: a cause of transient congenital hypothyroidism and delayed thyroid development. Eur J Endocrinol. (2004) 150:265–8. doi: 10.1530/eje.0.1500265 15012609

