# Supplementary material for: A novel bioassay for thyroid-blocking immunoglobulins
Source: Front Endocrinol (Lausanne). 2024 Oct 29;15:1463379. doi: 10.3389/fendo.2024.1463379 (PMC11554474; doi:10.3389/fendo.2024.1463379)
Supplement: Supplementary Formula 1 — Formula to calculate the percentage inhibition from relative light units in the Turbo™ TBI and Thyretain® TBI bioassays. x¯reference= average relative light units of the reference sample duplicate, x¯sample= average relative light units of the patient sample duplicate. [file Presentation1.pptx]

## Slide 1
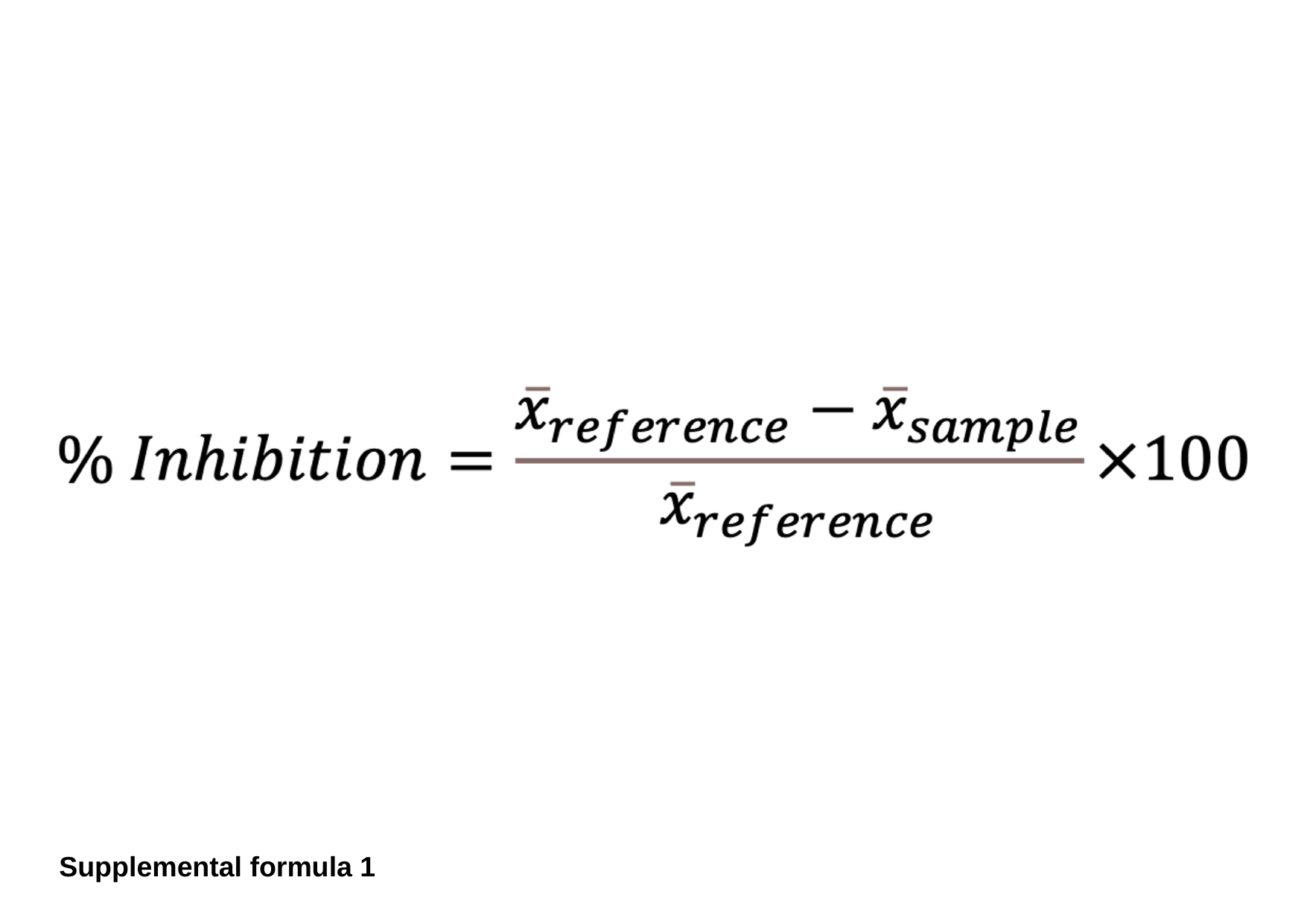

Supplemental formula 1

## Slide 2
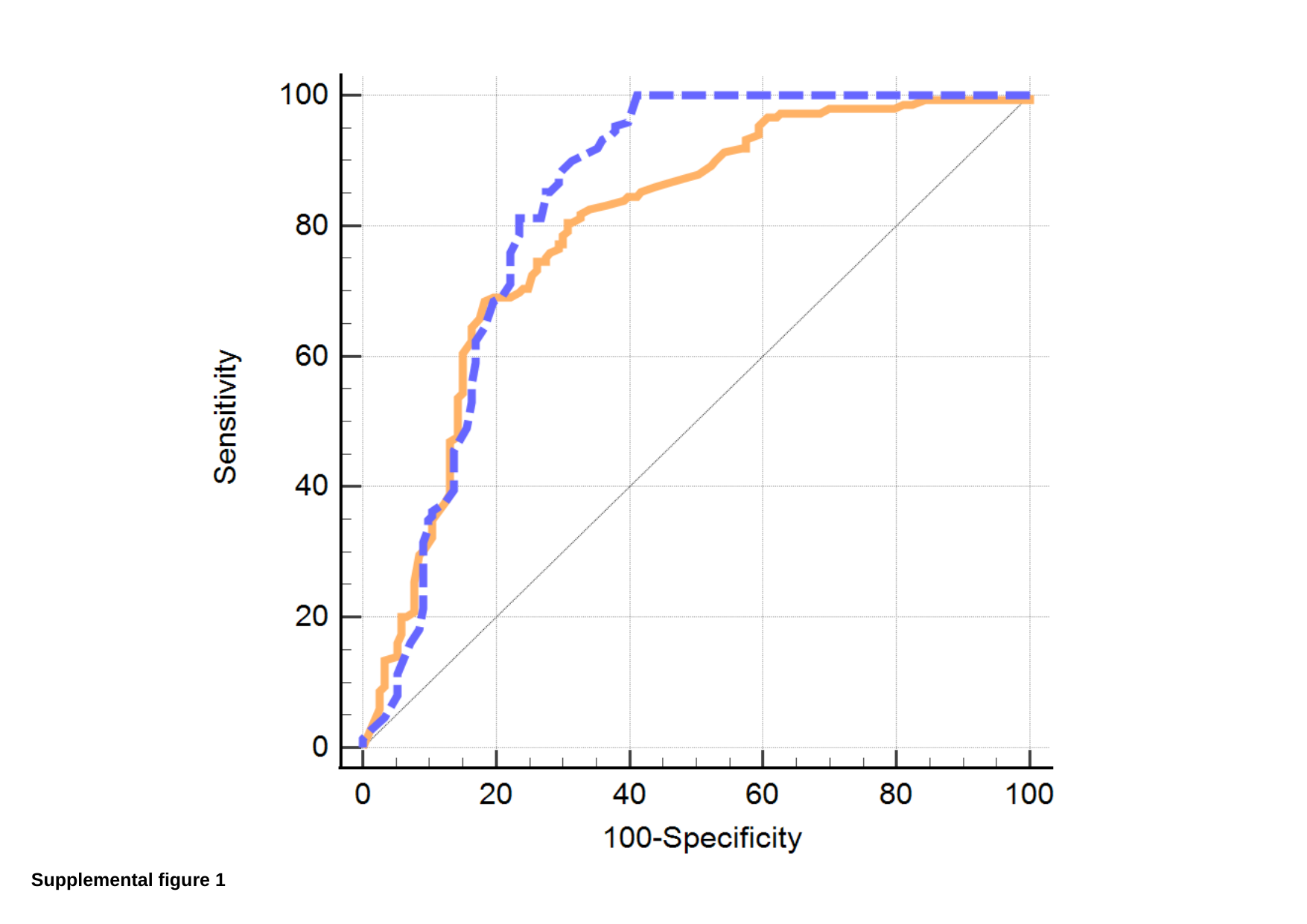

Supplemental figure 1

## Slide 3
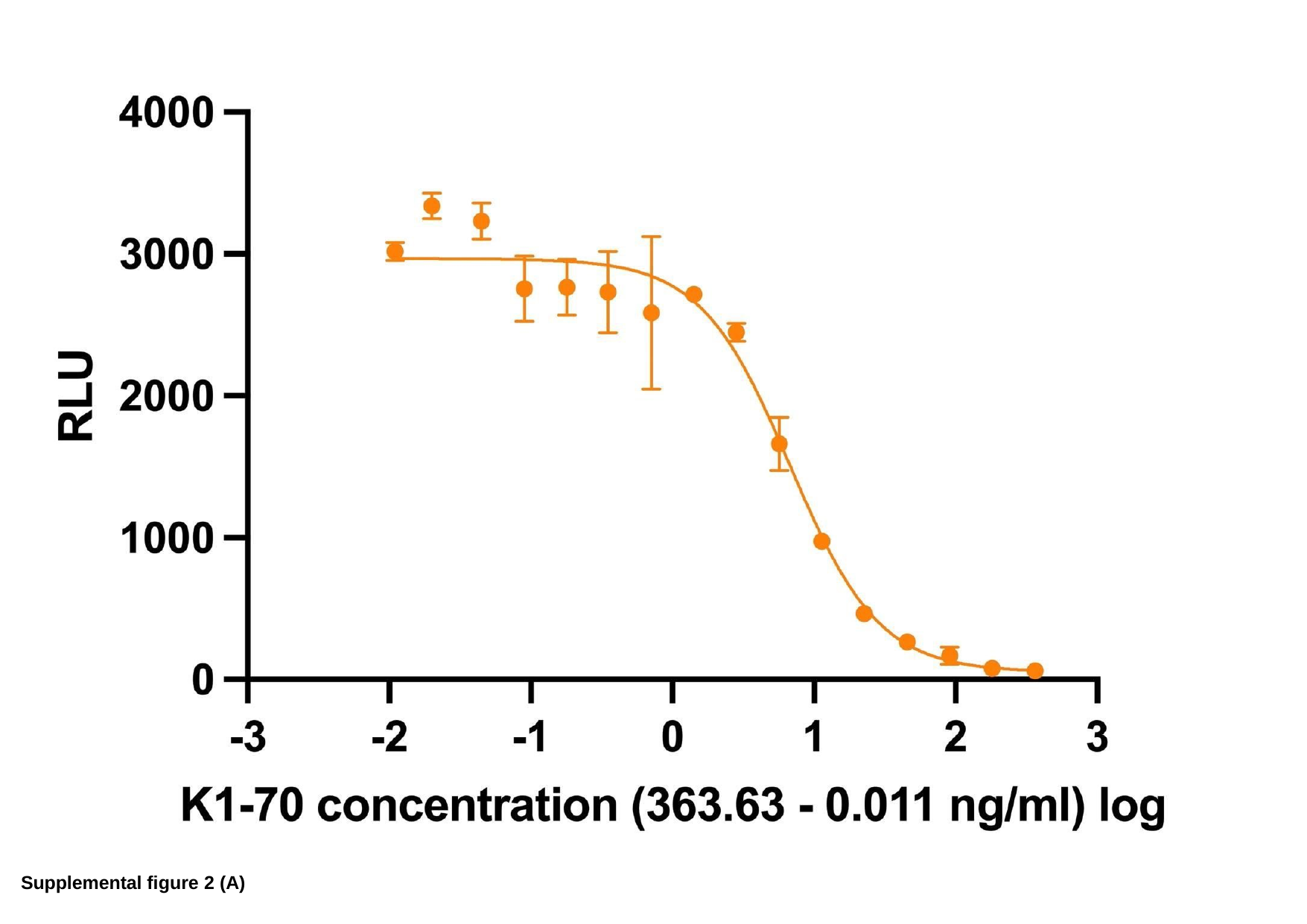

Supplemental figure 2 (A)

## Slide 4
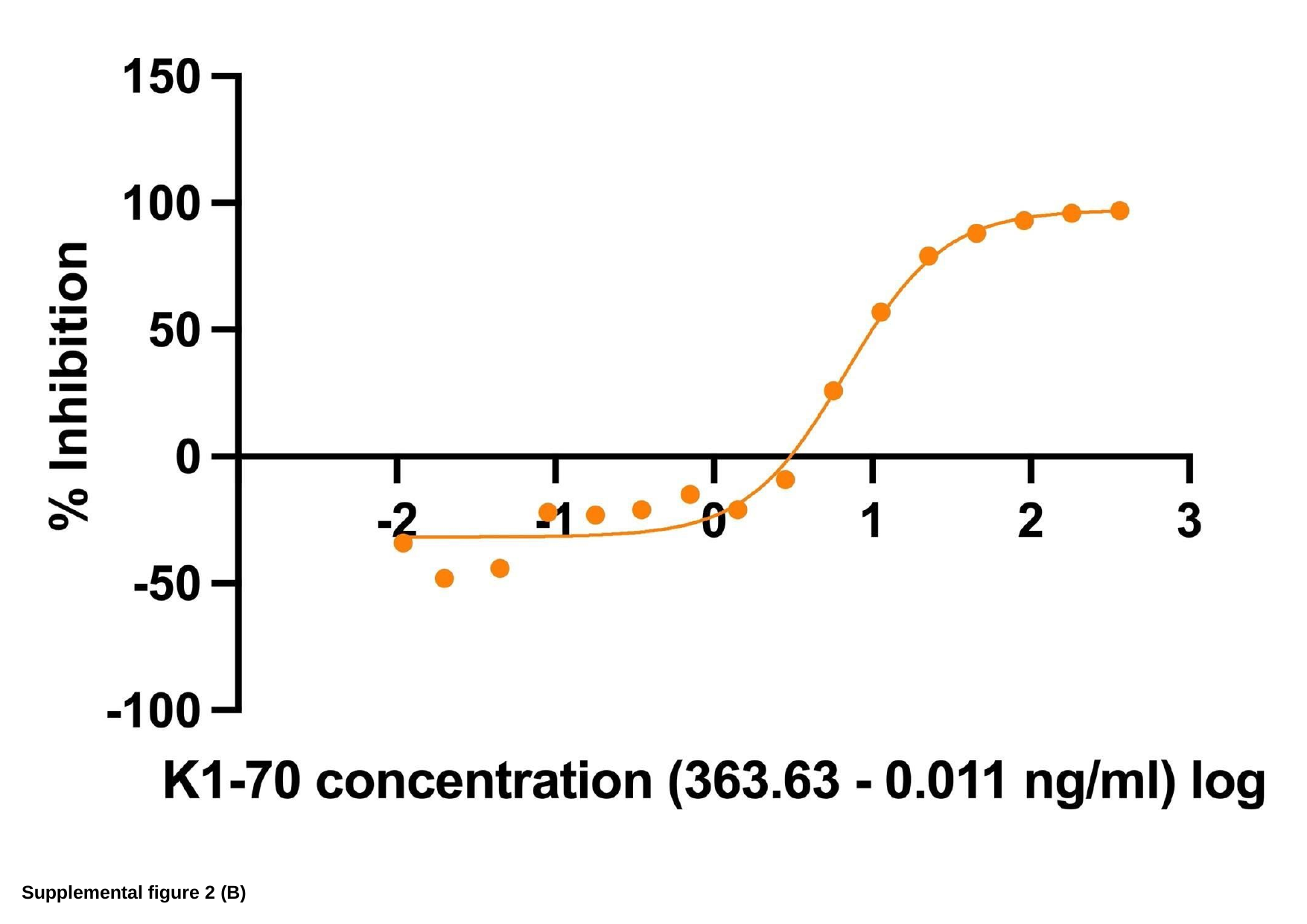

Supplemental figure 2 (B)

## Slide 5
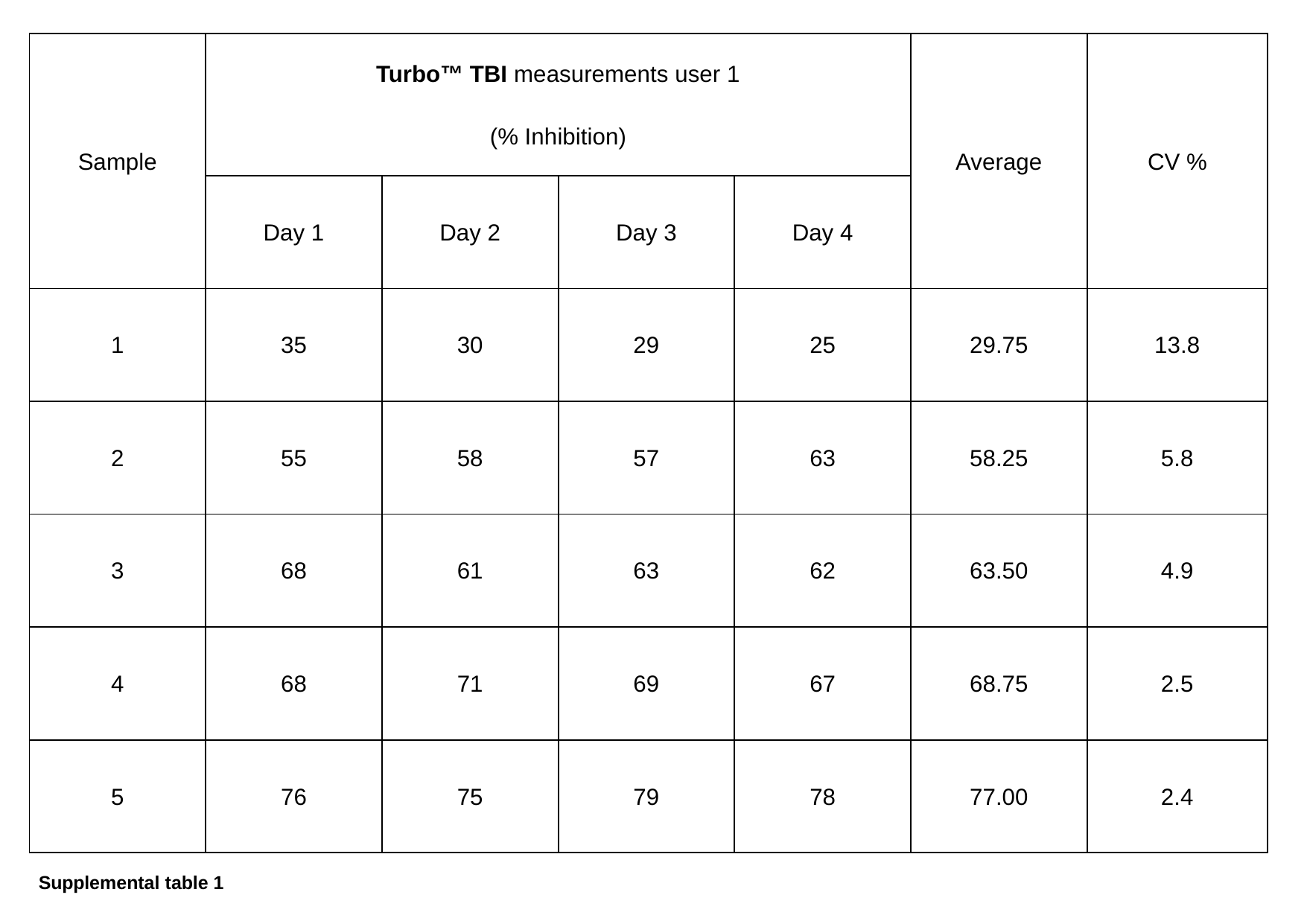

| Sample | Turbo™ TBI measurements user 1 (% Inhibition) | | | | Average | CV % |
| --- | --- | --- | --- | --- | --- | --- |
| | Day 1 | Day 2 | Day 3 | Day 4 | | |
| 1 | 35 | 30 | 29 | 25 | 29.75 | 13.8 |
| 2 | 55 | 58 | 57 | 63 | 58.25 | 5.8 |
| 3 | 68 | 61 | 63 | 62 | 63.50 | 4.9 |
| 4 | 68 | 71 | 69 | 67 | 68.75 | 2.5 |
| 5 | 76 | 75 | 79 | 78 | 77.00 | 2.4 |
Supplemental table 1

## Slide 6
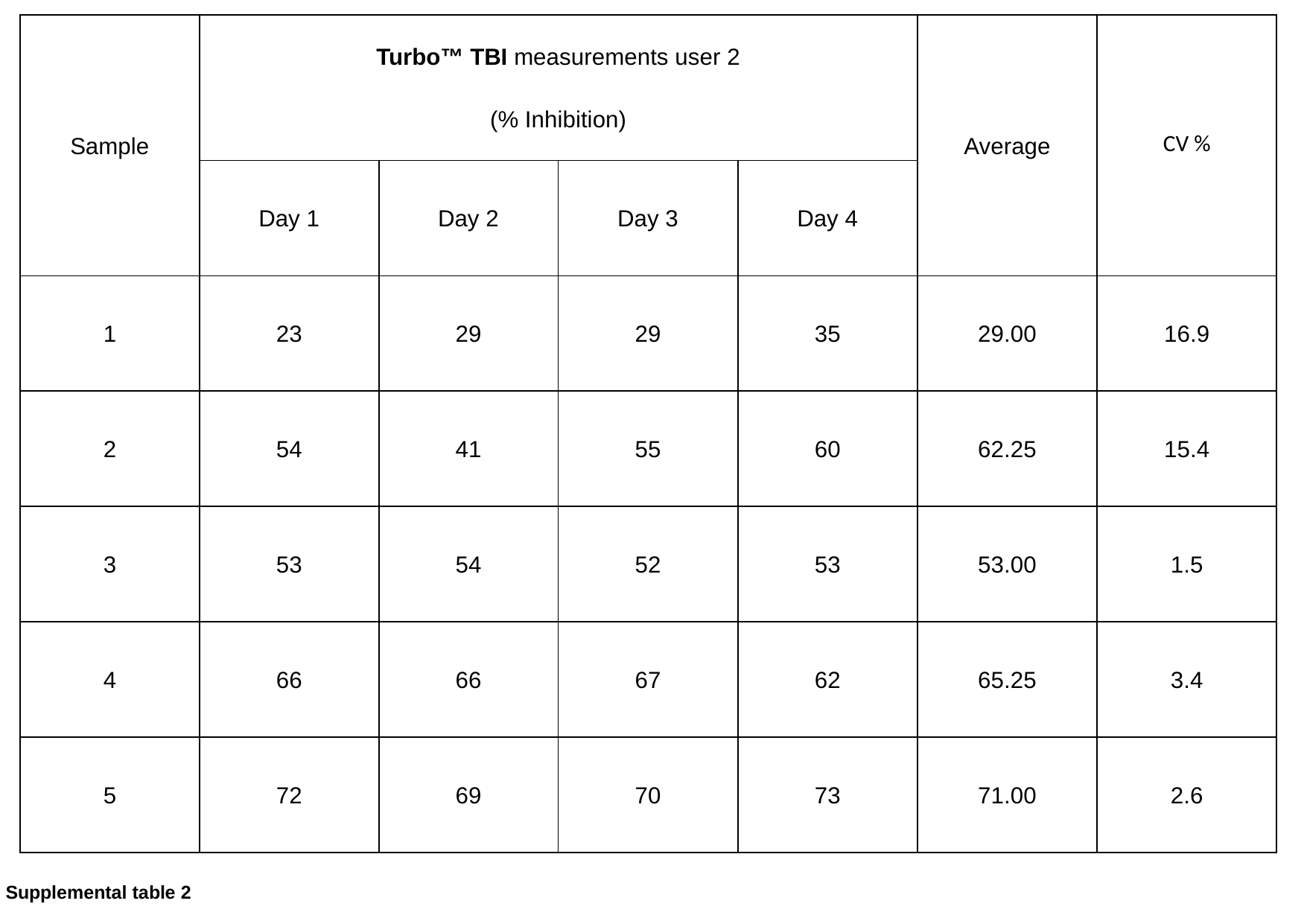

| Sample | Turbo™ TBI measurements user 2 (% Inhibition) | | | | Average | CV % |
| --- | --- | --- | --- | --- | --- | --- |
| | Day 1 | Day 2 | Day 3 | Day 4 | | |
| 1 | 23 | 29 | 29 | 35 | 29.00 | 16.9 |
| 2 | 54 | 41 | 55 | 60 | 62.25 | 15.4 |
| 3 | 53 | 54 | 52 | 53 | 53.00 | 1.5 |
| 4 | 66 | 66 | 67 | 62 | 65.25 | 3.4 |
| 5 | 72 | 69 | 70 | 73 | 71.00 | 2.6 |
Supplemental table 2

## Slide 7
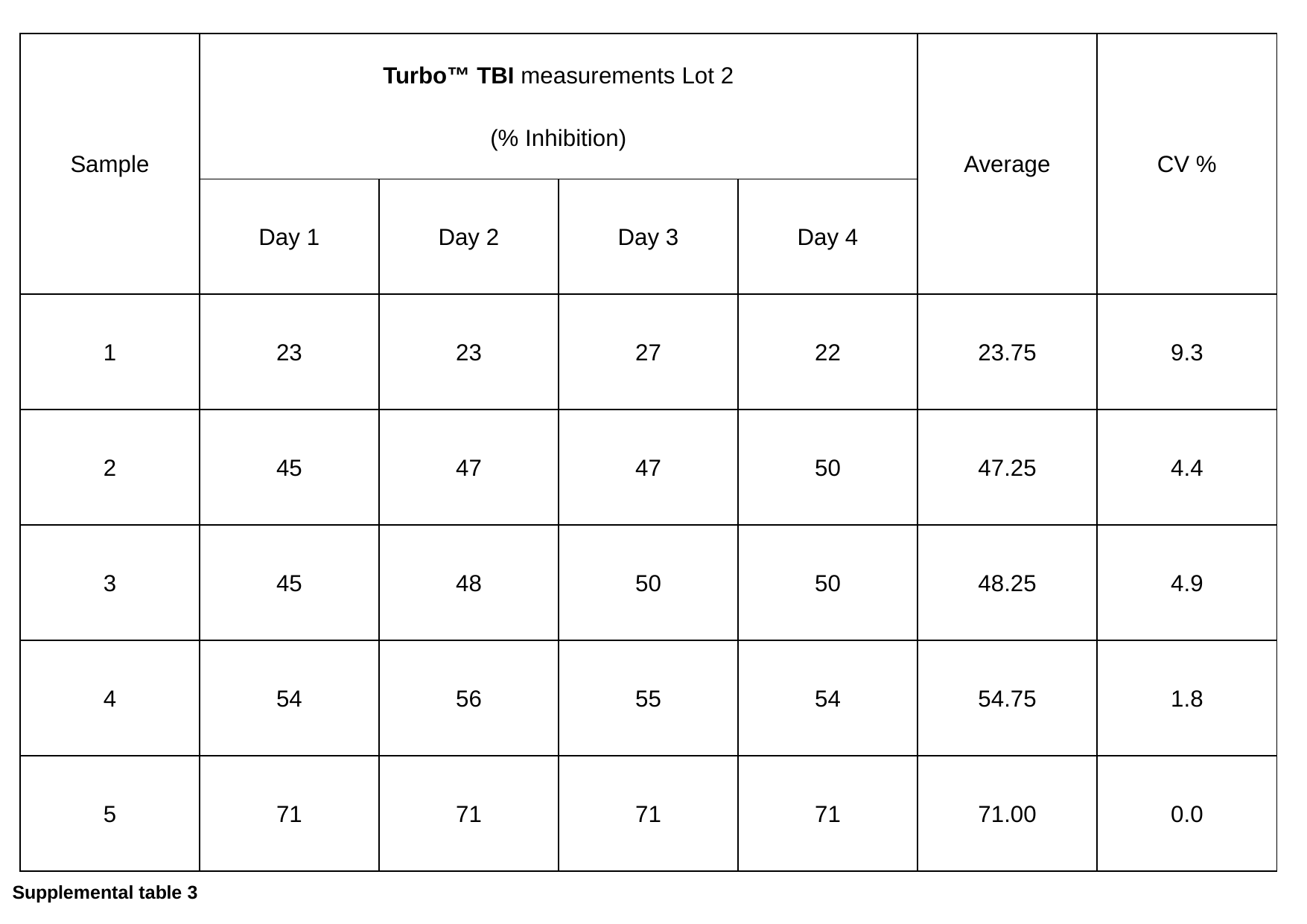

| Sample | Turbo™ TBI measurements Lot 2 (% Inhibition) | | | | Average | CV % |
| --- | --- | --- | --- | --- | --- | --- |
| | Day 1 | Day 2 | Day 3 | Day 4 | | |
| 1 | 23 | 23 | 27 | 22 | 23.75 | 9.3 |
| 2 | 45 | 47 | 47 | 50 | 47.25 | 4.4 |
| 3 | 45 | 48 | 50 | 50 | 48.25 | 4.9 |
| 4 | 54 | 56 | 55 | 54 | 54.75 | 1.8 |
| 5 | 71 | 71 | 71 | 71 | 71.00 | 0.0 |
Supplemental table 3

## Slide 8
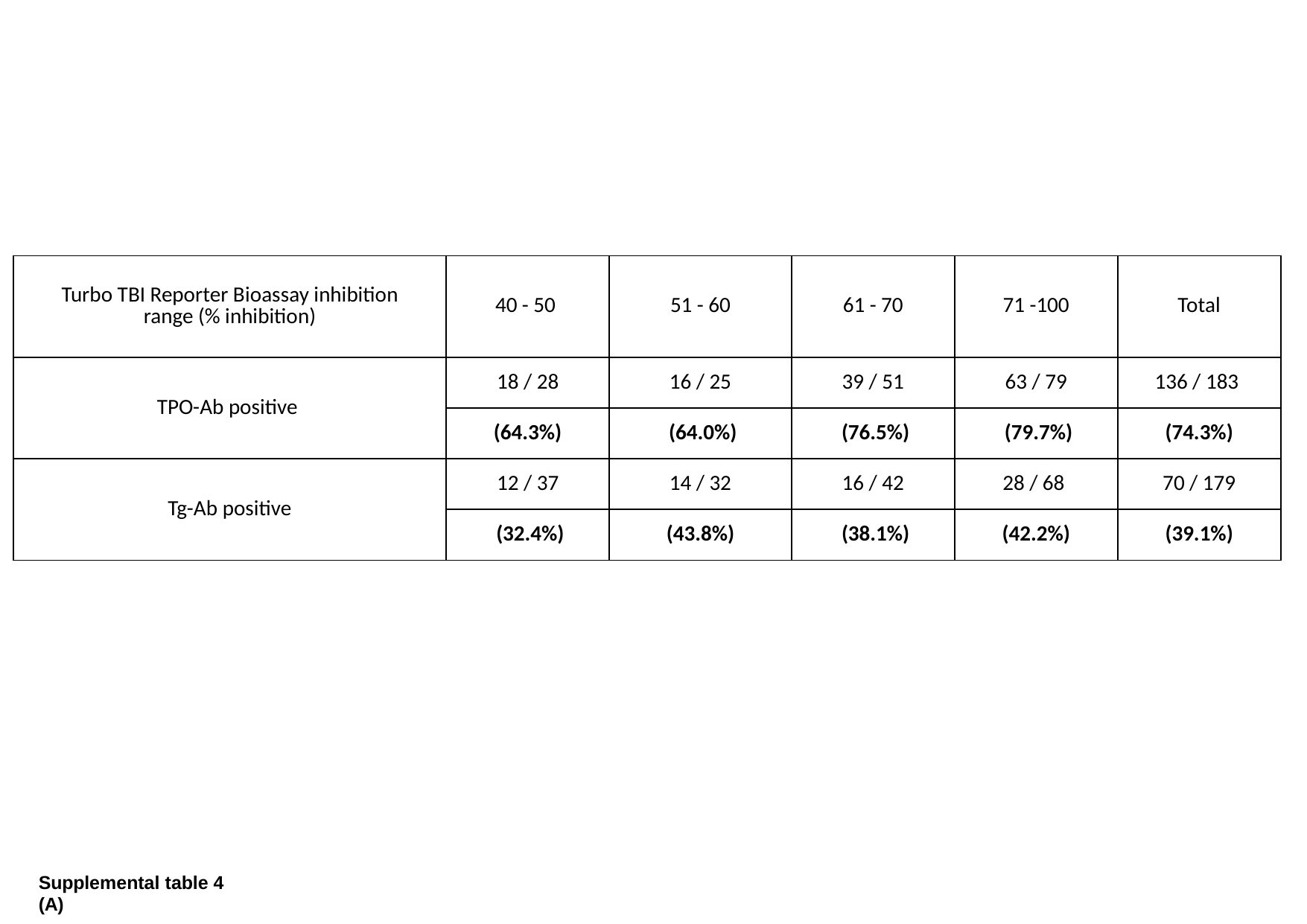

| Turbo TBI Reporter Bioassay inhibition range (% inhibition) | 40 - 50 | 51 - 60 | 61 - 70 | 71 -100 | Total |
| --- | --- | --- | --- | --- | --- |
| TPO-Ab positive | 18 / 28 | 16 / 25 | 39 / 51 | 63 / 79 | 136 / 183 |
| | (64.3%) | (64.0%) | (76.5%) | (79.7%) | (74.3%) |
| Tg-Ab positive | 12 / 37 | 14 / 32 | 16 / 42 | 28 / 68 | 70 / 179 |
| | (32.4%) | (43.8%) | (38.1%) | (42.2%) | (39.1%) |
Supplemental table 4 (A)

## Slide 9
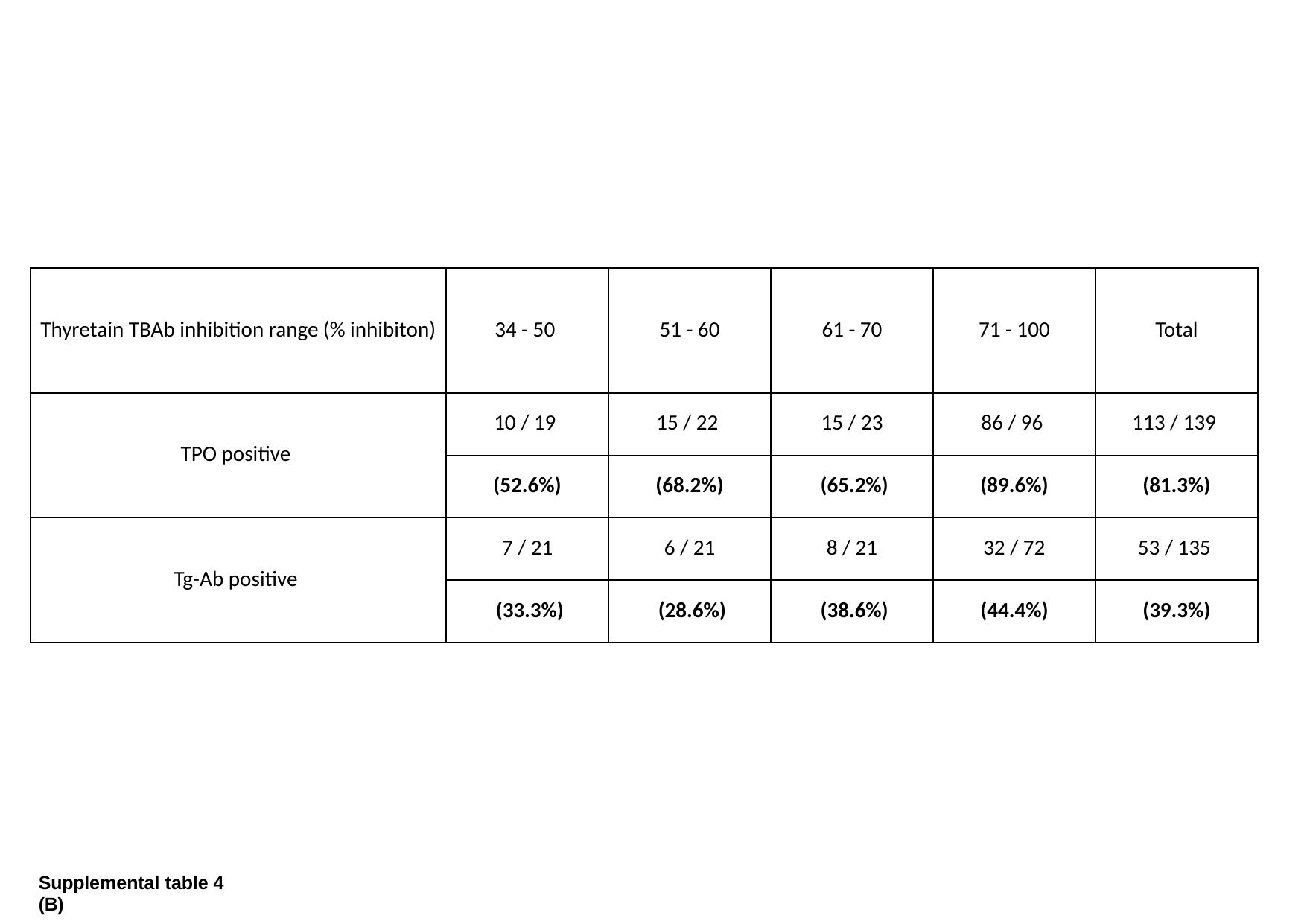

| Thyretain TBAb inhibition range (% inhibiton) | 34 - 50 | 51 - 60 | 61 - 70 | 71 - 100 | Total |
| --- | --- | --- | --- | --- | --- |
| TPO positive | 10 / 19 | 15 / 22 | 15 / 23 | 86 / 96 | 113 / 139 |
| | (52.6%) | (68.2%) | (65.2%) | (89.6%) | (81.3%) |
| Tg-Ab positive | 7 / 21 | 6 / 21 | 8 / 21 | 32 / 72 | 53 / 135 |
| | (33.3%) | (28.6%) | (38.6%) | (44.4%) | (39.3%) |
Supplemental table 4 (B)
